# Life-long Restructuring of 3D Genome Architecture in Cerebellar Granule Cells

**DOI:** 10.1126/science.adh3253

**Published:** 2023-09-07

**Authors:** Longzhi Tan, Jenny Shi, Siavash Moghadami, Bibudha Parasar, Cydney P. Wright, Yunji Seo, Kristen Vallejo, Inma Cobos, Laramie Duncan, Ritchie Chen, Karl Deisseroth

**Affiliations:** 1 Department of Neurobiology, Stanford University, Stanford, CA, 94305; 2 Department of Bioengineering, Stanford University, Stanford, CA, 94305; 3 Department of Chemistry, Stanford University, Stanford, CA, 94305; 4 Department of Chemical and Systems Biology, Stanford University, Stanford, CA, 94305; 5 Department of Biology, Stanford University, Stanford, CA, 94305; 6 Department of Pathology, Stanford University, Stanford, CA, 94305; 7 Department of Psychiatry and Behavioral Sciences, Stanford University, Stanford, CA, 94305; 8 Howard Hughes Medical Institute, Stanford, CA, 94305

## Abstract

The cerebellum contains most of the neurons in the human brain, and exhibits unique modes of development and aging. Here, by developing our single-cell 3D genome assay Dip-C into population-scale (Pop-C) and virus-enriched (vDip-C) modes, we resolved the first 3D genome structures of single cerebellar cells, created life-spanning 3D genome atlases for both human and mouse, and jointly measured transcriptome and chromatin accessibility during development. We found that while the transcriptome and chromatin accessibility of cerebellar granule neurons mature in early postnatal life, 3D genome architecture gradually remodels throughout life, establishing ultra-long-range intra-chromosomal contacts and specific inter-chromosomal contacts that are rarely seen in neurons. These results reveal unexpected evolutionarily-conserved molecular processes underlying distinctive features of neural development and aging across the mammalian lifespan.

## One-Sentence Summary

Human and mouse cerebellar granule cells continuously remodel transcriptome, epigenome, and 3D genome into distinctive formats throughout postnatal development and aging.

## Main Text:

Different cell types within the same organism can mature along highly-distinctive developmental and aging trajectories. At the molecular level, cell type–specific gene transcription can be orchestrated by diversity in genome architecture (folding of chromosomes in 3D) (1). Yet the genome architecture over the lifespan has not been elucidated, limiting our understanding of the life-spanning cellular dynamics of brain function and dysfunction.

Previously, using our single-cell 3D genome assay (diploid chromosome conformation capture, or Dip-C) (2–4), we found that cells in the mouse forebrain (cerebral cortex and hippocampus) undergo cell type–specific transformation in transcriptome and genome architecture during the first month of life (5). However, technological limitation hindered generalization of this finding across brain regions, species, and lifespan. Here we broaden our perspective along all three of these dimensions, by turning our focus across the neuraxis, from forebrain to hindbrain—specifically to the cerebellum, which contains ~80% of all neurons in the human brain. The cerebellum, a powerful yet compact processing unit that has expanded over evolution (6), exhibits unique characteristics including prolonged development after birth, malformation in autism (7), and degeneration during aging (8). Understanding genome dynamics of the cerebellum may provide insights into these unique features, as well as into motor control and cognition (9).

Prior work has revealed nuclear morphological features of cerebellar cells in vitro (10), but the 3D genome structures remains to be fully solved. Recently, chromosome conformation capture (3C/Hi-C) was performed on the adult cerebellum (11–13); however, a comprehensive, cross-species, single-cell 3D genome atlas of the developing and aging cerebellum is lacking. In addition, simultaneous analysis of transcriptome (14) and chromatin accessibility (15) in the cerebellum would provide additional valuable information. Here we show that the cerebellum undergoes an extraordinary, lifelong 3D genome transformation that is conserved between human and mouse, and is far greater in magnitude than forebrain (5), revealing genome rewiring as a potential molecular hallmark of aging.

### A 3D genome atlas of the developing and aging cerebellum

**Fig. 1.**
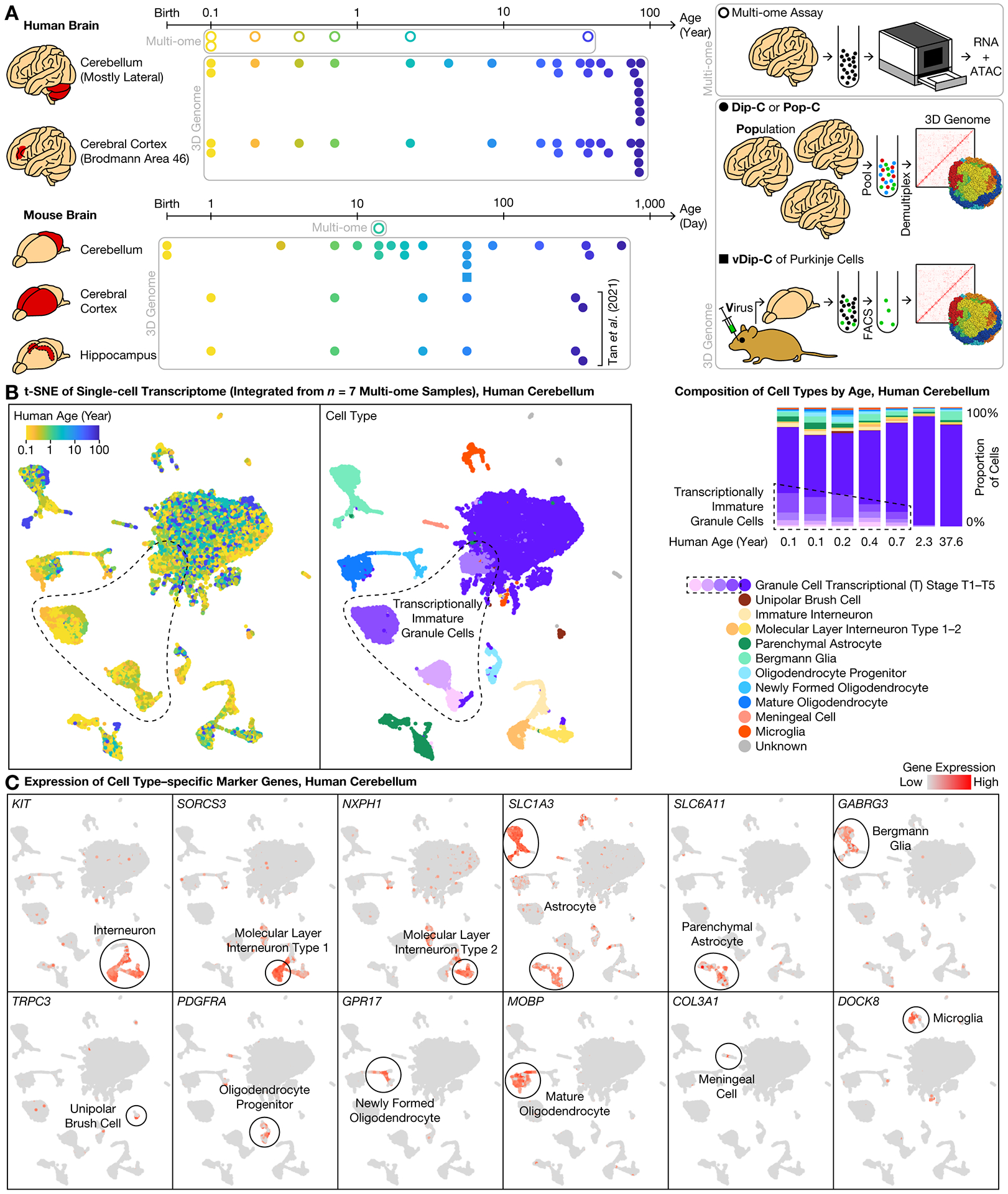
3D genome atlas across lifespan for human and mouse cerebellum with multi-ome atlas of postnatal development. **(A)** Study design. **(B)** Integrative transcriptome analysis of human multi-ome samples. **(C)** Representative expression profiles of marker genes.

Granule cells (the vast majority of cerebellar neurons) are generated between postnatal days P0–P21 in mouse, and between the third trimester of pregnancy and ~1 year after birth in human—much later than the same process in the cerebral cortex (5). During this period, granule cell progenitors divide and migrate from the external granular layer (EGL) to the internal granular layer (IGL), expanding >100-fold in number. Toward the other end of the lifespan, the cerebellum is also known to exhibit a slow epigenetic aging clock of DNA methylation (16). To explore the genomic underpinnings of this entire timeline, we created a 3D genome atlas extending across the human and mouse lifespan, alongside a multi-ome atlas focused on human development (Fig. 1A).

We first simultaneously profiled transcriptome and chromatin accessibility during postnatal development of the human cerebellum, sequencing 63,768 cells from 7 donors (17) (6 between the ages of 0.1–2.3 years, and 1 adult) and detecting a median of 645–1,617 genes (944–3,845 unique molecular identifiers (UMIs)) and 12–34 k assay for transposase-accessible chromatin (ATAC) fragments per cell from each donor (Table S1, Table S2). We additionally profiled a critical age in mouse—P14, when cells are present in both the EGL and the IGL—sequencing 7,182 cells and detecting a median of 618 genes (944 UMIs) and 22 k ATAC fragments per cell.

We then comprehensively profiled 3D genome architecture across the human and mouse lifespan, sequencing 11,207 cells (Fig. 1A). In human, we sequenced 5,202 cells from 24 donors (0.1–86 years) and obtained a median of 608,000 chromatin contacts per cell. Among these cells, 3,580 came from the cerebellum (chiefly lateral; vermis if lateral not available), and 1,622 from the cerebral cortex (Brodmann area (BA) 46 of the dorsolateral prefrontal cortex (DLPFC)) of the same donors (Table S2, Table S3, Table S4). In mouse, we sequenced 6,005 cells from cerebellum (birth to 21 months)—obtaining a median of 496,000 contacts per cell, and incorporated our prior dataset of 1,075 and 879 cells from mouse cerebral cortex and hippocampus, respectively (5).

### Transcriptionally immature granule cells in the newborn human cerebellum

We integrated the transcriptome dataset from all 7 human donors with LIGER (14, 18, 19) (Fig. 1B); modifying LIGER parameters did not affect conclusions (Fig. S1). Granule cells were the predominant cell type at all ages examined (median: 83%; range: 76–92%). Astrocytes (median: 7%; range: 3–11%) were composed of the cerebellum-specific Bergmann glia (4%) (18) and the typical parenchymal astrocytes (2%) (14, 15) (Fig. 1C). We further identified *GABRG3* as a specific marker for Bergmann glia (Fig. 1C). Other cell types included molecular layer interneurons (MLIs) (4%), oligodendrocytes (3%), microglia (1%), and unipolar brush cells (UBCs) (0.3%). Note that Purkinje cells were too rare to be reliably identified.

Unlike the case of mouse brain—wherein granule cells are almost entirely in the EGL at birth—the human cerebellum is already dominated at birth by IGL neurons. However, it remains unknown when and how human granule cells mature at the genomic level. We found that at birth, granule cells were subdivided into maturation stages by transcriptomic measures (Fig. 1B). In our integrated transcriptome data, granule cells existed in one mature form (termed transcriptional (T) stage T5) and several immature forms (T1–T4). T5 was the predominant form (99–100%) in the 2.3- and 37.6-year-old donors. In younger donors, however, T1–T4 made up a substantial fraction: 32–34% in the 0.1- and 0.2-year-olds, 23% in the 0.4-year-old, and 14% in the 0.7-year-old (Fig. 1B), revealing an abundance of transcriptionally immature granule cells.

**Fig. 2.**
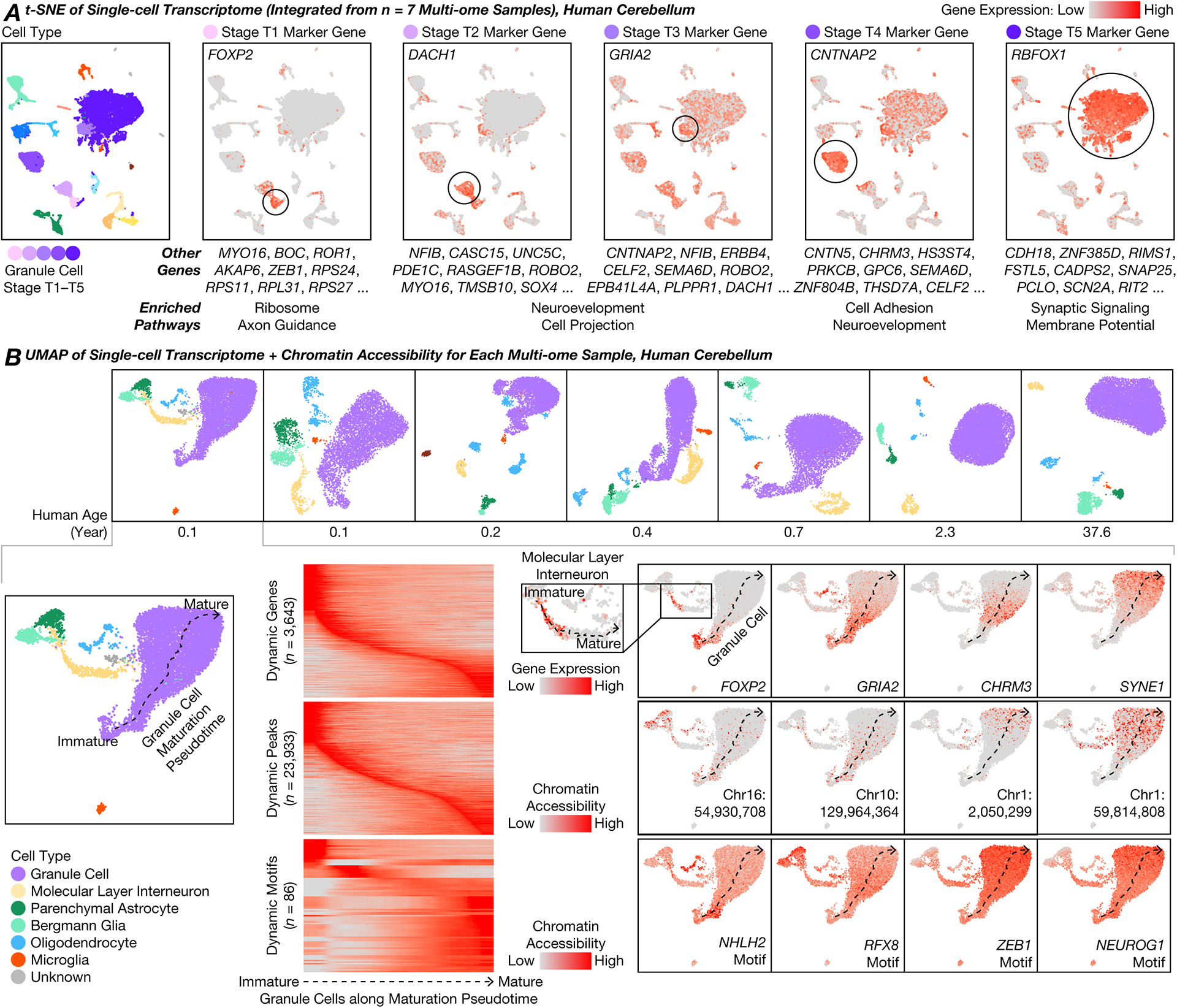
Simultaneous transcriptome and chromatin accessibility profiling revealed continuous maturation of cerebellar granule cells over the first postnatal year. **(A)** Stages of granule cell maturation, their marker genes (ranked by specificity), and enriched pathways (summarized for the top 100 genes). **(B)** Maturation pseudotime analysis of each sample.

T1–T5 granule cells expressed partially-overlapping sets of genes (Fig. 2A, Table S5). T1 was enriched for ribosomal subunits (FDR = 8 × 10^–67^; including *RPS24/11/27*, *RPL31/39/32*) as well as axon guidance-related genes (1 × 10^–4^; including *BOC*, *LAMA2*) and expressed *FOXP2*. T2 was enriched for neurodevelopmental genes (3 × 10^–6^; including *NFIB*, *UNC5C*) including additional genes related to morphogenesis of projections (3 × 10^–6^; including *ROBO2*, *MYO16*). T3 was also enriched for neurodevelopment (6 × 10^–12^; including *ERBB4*) and projection morphogenesis (1 × 10^–9^; including *SEMA6D*) and expressed *GRIA2*. T4 was enriched for cell adhesion (3 × 10^–14^; including *CNTNAP2/5*, *CNTN5*) and genes related to neurodevelopment (2 × 10^–9^; including *CHRM3*, *GPC6*). Finally, T5 was enriched for synaptic signaling genes (5 × 10^–16^; including *CADPS2*, *SNAP25*) and regulation of membrane potential (3 × 10^–11^; including *RIMS1*, *SCN2A*) and expressed *RBFOX1*. Correlated gene module analysis confirmed these genes and pathways (Fig. S3, Table S5).

### A continuum of granule cells and interneurons with maturing transcriptome and chromatin accessibility

For each donor, we jointly analyzed transcriptome and chromatin accessibility using ArchR (20) (Fig. 2B). Despite discrete appearances in LIGER (Fig. 1A), granule cells formed a continuous developmental pseudotime for each donor below the age of 1 year (Fig. S4, Fig. S6A). Dynamically expressed genes included many genes from LIGER analysis. Dynamically accessible transcription factor binding motifs included ASCL1/2 and NHLH1/2 (early), KLF11/14 and RFX2/3/4/8 (intermediate), and NEUROG1/2/3, NEUROD4/6, ZEB1, MEF2A/B/C/D, NFIA/B/X (late). The newborn human cerebellum was thus found to include a complex mixture of granule cells with continuously evolving transcriptomic and epigenomic states. We validated aspects of this continuum by re-analyzing published transcriptome-only data (14) (Fig. S6B). A continuum was also observed in mouse (Fig. S7) (14).

We observed a similar continuum of maturation in MLIs (Fig. S8). Immature MLIs were abundant at birth and vanished over age (64–71% in the 0.1- and 0.2-year-olds, 44% in the 0.4-year-old, 25% in the 0.7-year-old, 5% in the 2.3-year-old, and <1% in the adult) (Fig. 1B). In addition, immature MLIs and immature granule cells shared expression of many genes, such as *FOXP2* (Fig. 2B).

### 3D genome profiling of diverse populations and rare cells with Pop-C and vDip-C

We next focused on genome architecture. Cerebellar cells exhibit unique genome morphology, beginning with nuclear dimensions; granule cell nuclei are among the smallest in the brain (5–6 μm diameter), whereas Purkinje cells have large nuclei (~12 μm diameter) (21). During differentiation, cultured mouse granule cell progenitors reduce nuclear volume and spatially redistribute histone H3.3 (10). However, 3D genome structures of cerebellar cells have remains unclear and little is known about lifetime-spanning dynamics in vivo.

**Fig. 3.**
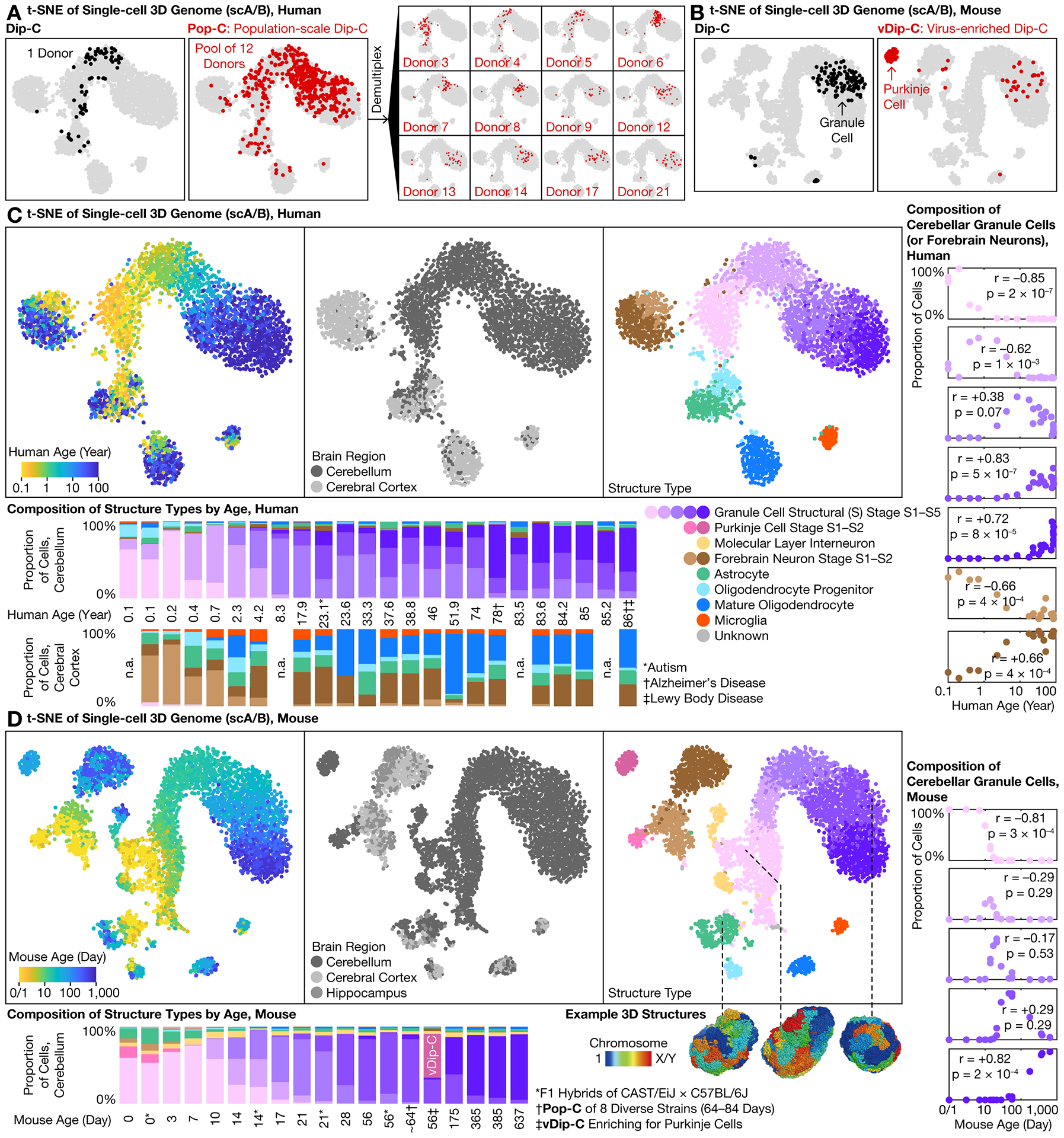
High-throughput, high-precision 3D genome profiling uncovered lifelong genome remodeling in the human and mouse cerebellum. **(A)** Pop-C method. **(B)** vDip-C method. **(C**–**D)** Cross-species 3D genome atlas for the developing and aging cerebellum (with cerebral cortex as counterpoint). Pearson’s r (and p-value) was calculated from logarithm of age.

To meet this challenge, we developed two 3D genome technologies. First, population-scale Dip-C (Pop-C) leveraged the whole-genome sequencing capability of Dip-C (2) to pool a large number of samples, and computationally demultiplexed (22) cells based on natural genetic variations, with high genomic coverage of 10–20% (Fig. 1A, Fig. 3A). We validated Pop-C by computationally pooling known samples (accuracy: 672/672 = 100%; Fig. S10).

In the simplest case, many of our mouse samples were a pool of males and females, which we demultiplexed based on the ratio of reads between X chromosome and autosomes (Fig. S9). In a more complex case, we pooled one mouse each from the 8 founder strains of the JAX Diversity Outbred (DO) collection (23), and demultiplexed based on known single-nucleotide polymorphisms (SNPs) (Table S4). In the most challenging case, we pooled 3–13 unrelated human individuals and demultiplexed them (22) based on common SNPs among populations, without prior knowledge about donor genotypes (Fig. 3A). Pop-C was thereby shown to provide a robust method for profiling single-cell 3D genome at scale (Fig. S11).

The second method developed here, virus-enriched Dip-C (vDip-C), enabled genomic profiling of rare cell populations without use of transgenic mouse lines (Fig. 1A, Fig. 3B). We used a single viral vector containing a cell type–specific promoter, an ultra-bright, fixation-resistant, monomeric fluorescent protein (24), and a nuclear membrane localization sequence (25) (Fig. S12) and administered it to wild-type mice (26) via retro-orbital injection of adeno-associated virus (AAV) (27, 28).

We used vDip-C to solve the 3D genome structures of Purkinje cells (Fig. 3B). Although Purkinje cells are abundant at birth (P0), they quickly become outnumbered by granule cells (Fig. 3D). To isolate this rare (<0.5%) cell type from adults, we constructed a vDip-C vector with a Purkinje cell–specific promoter (*Pcp2*) (29), administered the viral vector to wild-type mice, and isolated nuclei by fluorescence-activated cell sorting (FACS) (Fig. S12).

### Lifelong 3D genome transformation of granule cells

We created a high-resolution, cross-species single-cell 3D genome atlas and resolved 3D genome structures for a subset of cells (from F1 hybrid mice) (Fig. 1A, Fig. 3C, Fig. 3D). Similar to our previous studies (2, 3, 5), single-cell chromatin A/B compartment (scA/B) analysis revealed 3D genome structure types corresponding to diverse cerebellar cell types—including granule cells, astrocytes, oligodendrocytes, and microglia in both species, as well as MLIs and Purkinje cells in mouse. Replicates yielded reproducible scA/B and contact patterns (Fig. 3A, Fig. S11, Fig. S21). Note that 3 of our 24 donors were diagnosed with autism, Alzheimer’s disease, and/or Lewy body disease; excluding them did not affect our conclusions (Fig. S28).

Granule cells exhibited by far the most dramatic structural transformation. Granule cells of both species were born with an immature structure type, termed structural (S) stage S1, that resembled forebrain neurons (Fig. 3C, Fig. 3D, Fig. S14, Fig. S19). As the cerebellum developed and aged, granule cells continuously and progressively evolved into new structure types S2–S5, which increasingly differed from forebrain neurons (Fig. S14, Fig. S19). This transformation was the primary source of scA/B variations (the first principal component (PC)) and could be visualized regardless of the analysis method (Fig. S15, Fig. S16).

In human, abundances of S1–S5 peaked around the ages of 0.2, 1, 10, 30, and 80 years, respectively, although considerable between-donor variability was observed (Fig. 3C). This age distribution suggested that S2–S5 likely all corresponded to T5. In mouse, S1–S5 peaked around P3, P14, P21, P56, and P365 (Fig. 3D); note that within S5, 3D genome continued to mature between P365/385 (~12 months) and P637 (~21 months) (Fig. S17). These data reveal a 3D genome aging clock of large architectural transformation in a mostly post-mitotic cell type.

### Ultra-long-range intra-chromosomal contacts and specific inter-chromosomal contacts in granule cells

**Fig. 4.**
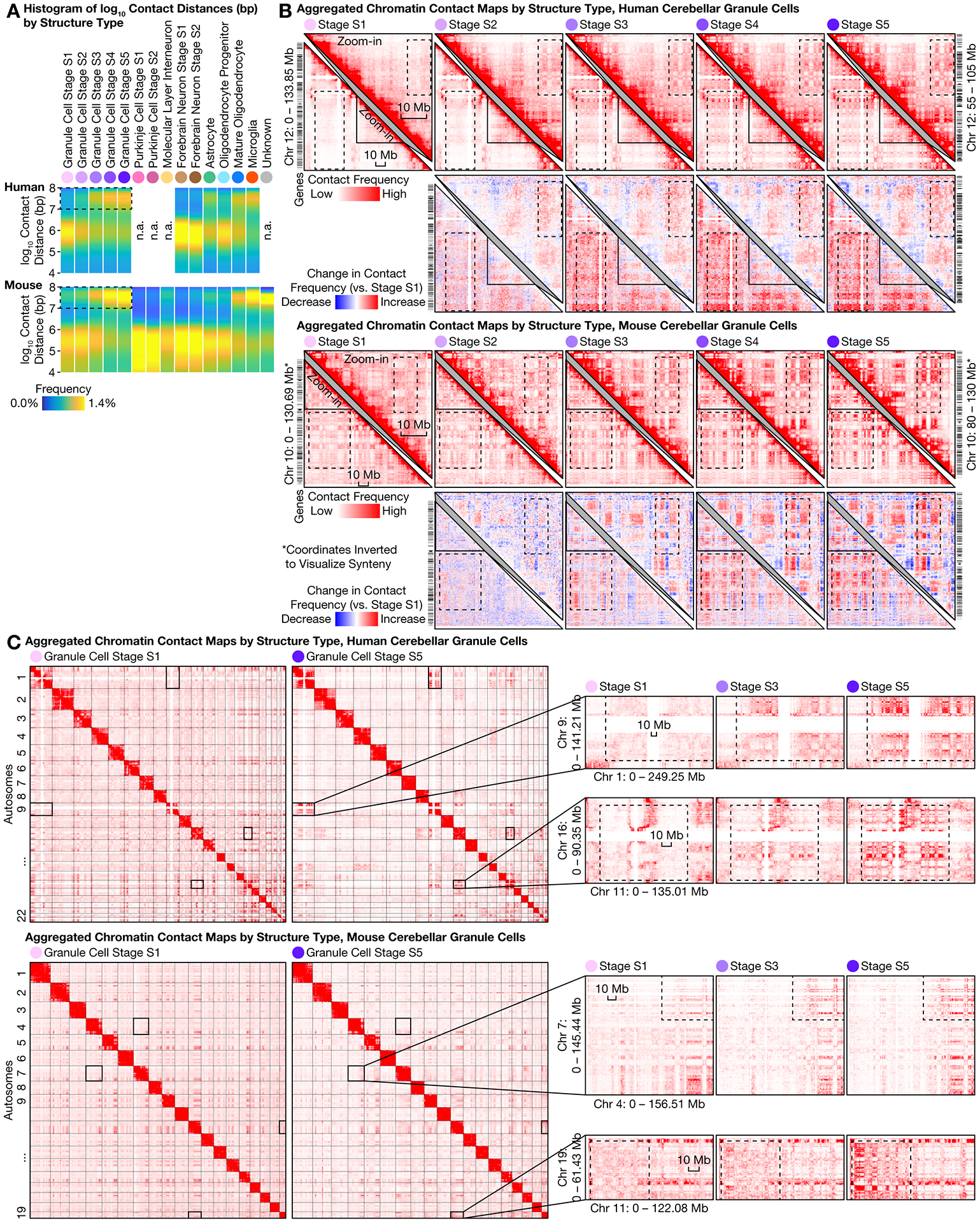
Cerebellar granule cells formed ultra-long-range intra-chromosomal contacts and specific inter-chromosomal contacts during development and aging. **(A)** Distribution of genomic distances of chromatin contacts. **(B)** Aggregated contact maps for an example chromosome and a zoomed-in region (upper right triangles). Zoomed-in regions are homologous. Dashed boxes highlight prominent changes. Bin size: 250 kb. **(C)** Aggregated inter-chromosomal contact maps. Bin sizes: 6 Mb (human); 5 Mb (mouse); 500 kb (zoom-in).

The most prominent architectural changes in granule cells were the emergence of ultra-long-range (10–100 Mb) contacts, which had been thought largely restricted to non-neuronal cells (13, 30) with the exception of mouse rod photoreceptors (3) (Fig. 4A). From S1–S5, the fraction of contacts that were ≥10 Mb steadily increased from (19 ± 4)% to (33 ± 3)% in human (mean ± s.d.; two-sided U test p = 7 × 10^–205^), and from (19 ± 6)% to (34 ± 2)% in mouse (p < 10^–300^), far greater in magnitude than for forebrain neurons (from (15 ± 4)% to (16 ± 5)% during human development, p = 0.038, and from (11 ± 4)% to (13 ± 3)% in mouse, p = 4 × 10^–36^) and Purkinje cells (from (9 ± 1)% to (10 ± 2)%. p = 2 × 10^–5^) (Fig. S18).

This progression in granule cells might be partly driven by their small nuclear size. The abundance of ultra-long-range contacts resembled that seen in non-neuronal cells such as microglia ((34 ± 3)% in both species) and mature oligodendrocytes ((29 ± 4)% in human, (27 ± 5)% in mouse), all of which have similarly small nuclei. However, these cell types differed substantially in the genomic loci that formed such contacts (Fig. S19) and in scA/B profiles (Fig. S14), suggesting that nuclear size was not the only driving force.

Granule cells’ redistribution of intra-chromosomal contacts was accompanied by highly specific inter-chromosomal contacts. We found increasing interactions among certain human chromosomes—most prominently within a hub of Chr 1, 9, 11, 14, 15, 16, 17, 21, and 22, and between chromosome pairs such as Chr 2/9, 4/14, 8/11, 13/20; meanwhile, Chr 12 weakened its interactions with the hub (Fig. 4C, Fig. S20). Both ultra-long-range intra-chromosomal contacts (Fig. 4B) and inter-chromosomal contacts often involved large stretches of the heterochromatic compartment B, interleaved with small stretches of the euchromatic compartment A (also known as “mega-loops/enhancers” (31)). We also observed inter-chromosomal contacts in mouse (Fig. 4C); for example, mouse Chr 7 gained interactions with 4, 5, 11, 17, and 19. These results highlight another example of conserved, specific inter-chromosomal interactions beyond prior discoveries in nasal tissue (3, 32–34).

### Life-spanning scA/B changes associated with granule cell–specific marker genes

**Fig. 5.**
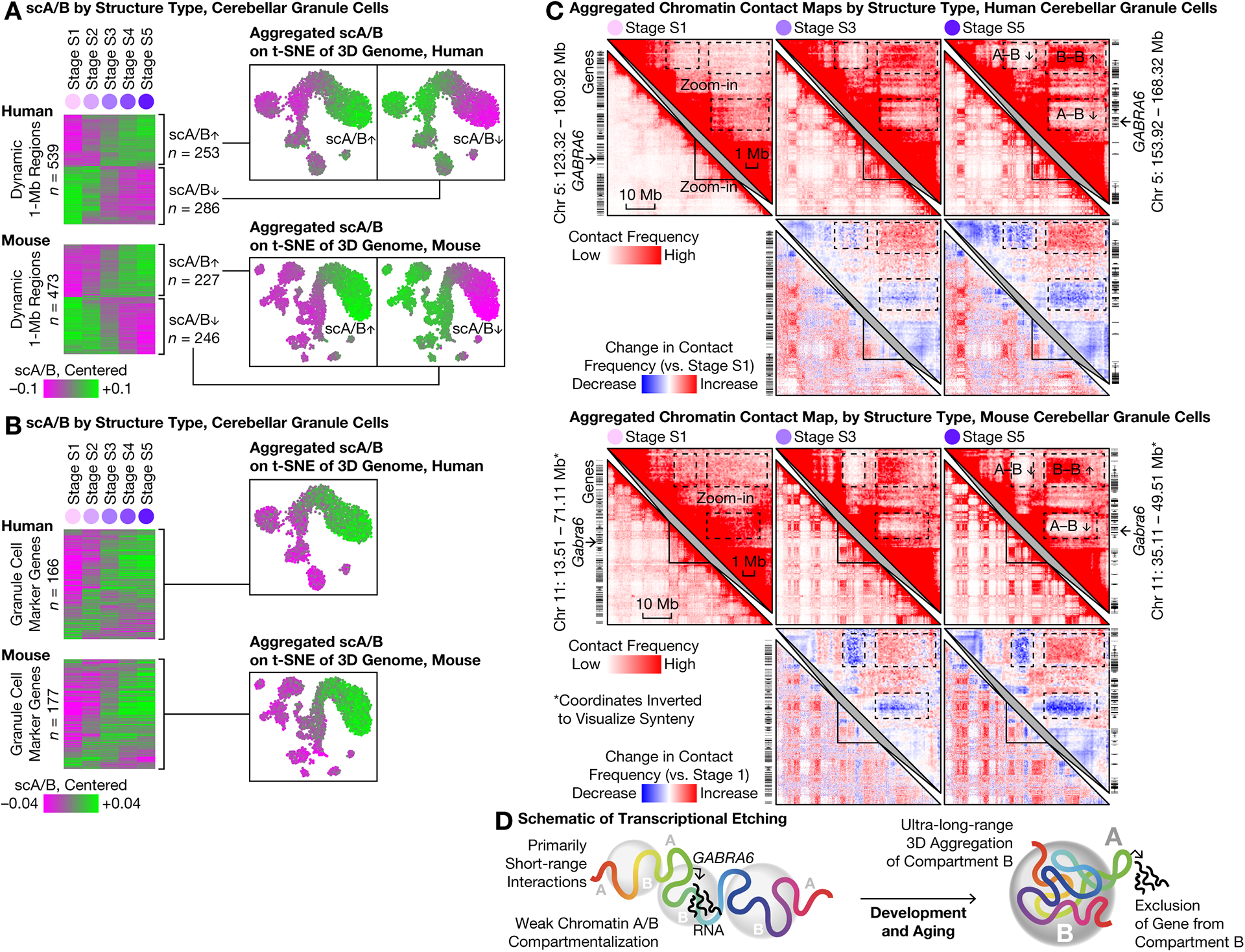
Lifelong maturation of chromatin A/B compartments was associated with cerebellar granule cell–specific genes. **(A)** Mean scA/B of each dynamic 1-Mb region at each stage (left). Rows are ordered by hierarchical clustering; the two clusters were visualized on t-SNE plots (right). As in (5), scA/B calculation excludes contacts within each region and thus primarily reports on long-range interactions. **(B)** Mean scA/B of each 1-Mb region harboring granule cell–specific marker genes (14) at each stage (left), with aggregated scA/B shown on t-SNE plots (right). **(C)**. Aggregated contact maps for an example gene. Bin size: 100 kb. **(D)** Schematic of transcriptional etching.

We previously showed that scA/B generally correlates with cell type–specific gene expression, although discordance can be observed at the single-gene level and regarding temporal dynamics (3, 5), and it has remained unclear how scA/B interacts with gene expression during aging. In granule cells, we found the predominant mode of scA/B changes to be progressive up- or down-regulation. We calculated the mean scA/B of each 1-Mb genomic region at S1–S5, and identified the top 20% dynamic regions. In both species, these ~500 dynamic regions either gradually increased or decreased in scA/B across S1–S5 (Fig. 5A).

We examined genomic regions that harbored conserved marker genes of mature granule cells (14). Expression of these ~200 genes began around birth, when T5 emerged. In contrast, on average, these loci gradually increased scA/B throughout life (Fig. 5B, Fig. S22). For example, *GABRA6* gradually lost contacts with 2 nearby heterochromatic regions (which steadily gained contacts with each other) over the lifespan (Fig. 5C), and consequently increased scA/B until S3 (~10 years) in human (two-sided U test p = 9 × 10^–11^ from S1–S2, 1 × 10^–5^ from S2–S3) and until S4 (~P56) in mouse (p = 2 × 10^–22^ from S1–S2, 6 × 10^–10^ from S2–S3, 9 × 10^–6^ from S3–S4)—persisting well after transcriptional up-regulation at ~0.5 years and ~P10, respectively (14). Down-regulated genes on average exhibited relatively unchanged scA/B, consistent with previous observations (3). In conclusion, increased transcription may continue to etch 3D genome structure long after initial gene activation (Fig. 5D) (5).

### Robust 3D genome maturation despite functional perturbations

To test robustness of this genome restructuring, we explored functional perturbation of chromatin remodeling (Fig. S26). Using bulk Dip-C, we observed little effect on 3D genome maturation in mice with clinically-relevant heterozygous deletion of *Arid1b* (35) or *Chd8*, although we cannot rule out more subtle differences. Granule cell–specific, homozygous deletion of *Chd4* caused moderate 3D changes (12); however, these changes had little overlap with (and were much smaller than) our observed architectural maturation.

## Discussion

Once born, most neurons must last for a lifetime; however, we know little about how underlying genomic information may be structurally organized. Here we discovered unique genome architecture in cerebellar granule cells: ultra-long-range contacts that are uncommon in neurons, specific inter-chromosomal contacts reminiscent of those in nasal tissue (3), and remodeling over decades that may be stabilized by cell type–specific gene transcription. We showed that mouse is an excellent animal model of this process, despite substantial differences from human beings in lifespan.

We provided mechanistic insights into the principles of this reorganization. For example, both granule cells and Purkinje cells lack neuron-specific non-CpG DNA methylation (13), revealing that non-CpG methylation was neither required for our previously discovered, neuron-specific radial genome movement (5)—which we observed in both cell types (Fig. S27) (36), nor required for suppressing ultra-long-range contacts.

A potential function of this architecture might be to manage space and energy expenditure. Our brains are 80% cerebellar granule cells by neuron number. If each granule cell consumed the same volume and energy as a typical neuron in the cerebral cortex, metabolic costs could become prohibitive. Consistent with this idea, granule cells are quiet by firing rate (~0.1 Hz) (37), in contrast to Purkinje cells (~50 Hz) (38). Granule cells might therefore have adopted an energy-saving state: physiologically, transcriptionally, and architecturally. It is worth noting that cerebellar and hippocampal granule cells adopt different structural strategies, despite similar nomenclature. Hippocampal granule cells were more similar to other forebrain neurons than to cerebellar granule cells (Fig. S14) and have larger nuclei (9–10 μm) (39, 40), although both are similarly inactive (firing rate 0.1–0.2 Hz) (41). It remains to be determined how granule cells in the olfactory bulb organize their genome.

More broadly, this approach showcases how life-spanning 3D genome profiling of a complex, living tissue can provide unprecedented dimensions of information. This lifelong structural transformation may point the way to new therapeutic targets for developmental and aging-related disorders. Wide application of the 3D genome technologies developed here to many brain regions and tissues of the body may contribute to solving longstanding challenges such as dissecting the genetic basis of inter-individual variability, characterizing ultra-rare cell types, and revealing the full diversity and dynamics of 3D genome organization across the life of mammals.

This study has certain important limitations. For example, we used frozen human samples, which might differ from fresh samples. We also note that vDip-C does not apply to human; however, human Purkinje cells could alternatively be isolated by flow cytometry based on size. Finally, future work will be required to test functional relationships between structural and transcriptional changes.

## Supplementary Material

Supplementary Materials

Table S1

Table S3

Table S2

Table S5

Table S4

Table S6

## Data Availability

Raw and processed data is available under the BioProject PRJNA933352 (https://www.ncbi.nlm.nih.gov/bioproject/?term=PRJNA933352). Code is available from GitHub (https://github.com/tanlongzhi/dip-c). The vDip-C vector will be available from Addgene.
